# Biofilm and its implications postfracture fixation: All I need to know

**DOI:** 10.1097/OI9.0000000000000107

**Published:** 2021-06-15

**Authors:** Nikolaos K. Kanakaris, Peter V. Giannoudis

**Affiliations:** a Academic Department of Trauma and Orthopaedics, School of Medicine, University of Leeds; b NIHR Leeds Biomedical Research Center, Chapel Allerton Hospital, Leeds, United Kingdom

**Keywords:** bacteria, biofilm, orthopaedic trauma infections, planktonic state, sonication

## Abstract

Biofilm represents an organized multicellular community of bacteria having a complex 3D structure, formed by bacterial cells and their self-produced extracellular matrix. It usually attaches to any foreign body or fixation implant. It acts as a physical protective barrier of the bacteria from the penetration of antibodies, bacteriophages, granulocytes and biocides, antiseptics, and antibiotics. Biofilm-related infections will increase in the near future. This group of surgical site infections is the most difficult to diagnose, to suppress, to eradicate, and in general to manage. Multispecialty teams involved in all stages of care are an effective way to improve results and save resources and time for the benefit of patients and the health system. Significant steps have occurred recently in the prevention and development of clever tools that we can employ in this everlasting fight with the bacteria. Herein, we attempt to describe the nature and role of the “biofilm” to the specific clinical setting of surgical site infections in the field of orthopaedic trauma surgery.

## 1 Introduction

The forecast for orthopaedic trauma surgery and the usage of fracture fixation implants suggests a significant increase (>30%) within the next 10 years, exceeding, as a market size, the $9.5 billion mark by 2025.^[1]^ On a global scale, in parallel with the high energy orthopaedic trauma that continues to increase, the growing prevalence of osteoporosis and the rising number of sports injuries, all contribute to the expected spur on the demand for fixation implants. Moreover, advances in the design of implants, their user-friendliness, and the globalization of surgical education also contribute to these large predictions.^[2,3,4]^

At the same time, the incidence of complications and adverse effects of this type of surgical intervention is also increasing.^[5,6]^ One of the most devastating complications following orthopaedic trauma surgery is the development of a deep surgical site infection (SSI).^[7]^ SSIs are mostly attributed to the perioperative contamination of the surgical field, which may declare itself at an early (first 2 months), delayed (3 months to 2 years), or late period (>2 years). Less frequently, it can also occur following secondary delivery of pathogens to the surgical site due to unrelated bacteraemias.^[8,9]^ Interestingly, a number of associated risk factors have been explored including the severity of the initial soft tissue injury and bony trauma, patient comorbidities (e.g., diabetes, obesity, alcohol consumption), and the prolonged use of external fixation devices prior to internal fixation among others.^[10,11]^

The spectrum of bacterial flora responsible for these infections has been extensively investigated in numerous series. In reality, any bacterial or even mycobacterial or fungal microorganism can produce an SSI. The most common are members of the *Staphylococcus aureus* family, gram-negative rods, or combinations of species (polymicrobial infections).^[12,13,14]^

The contemporary understanding is that the same bacteria can exist in 3 distinct phenotypic states at the infected site, that is, “free-floating” planktonic state”; “attached” sessile state; and “quasi-sessile” state. There is a biological continuum between these different states of phenotypic and metabolic homeostasis. The details, as well as the importance of the sequential transition between these different phenotypes, became apparent during the last 30 years. Scientists realized that bacterial communities, similar to interacting and attaching onto surfaces of the natural environment (firstly described by engineers on sanitation and water filtration systems), can do the same on any implantable device inside the human body.^[15]^ In 1940, this type of a bacterial formation was observed at the oral cavity and was linked to a specific clinical condition i.e. the dental plaque.^[16]^ In the early 80s, clinicians started using the term “biofilm,” discovering gradually its relevance to a number of chronic diseases, and infections of the soft and bone tissues.^[17]^

Herein, we attempt to describe the nature and role of the “biofilm” to the specific clinical setting of surgical site infections in the field of orthopaedic trauma surgery.

## 2 Definitions

The term “biofilm” describes an organized multicellular community of bacteria that is surrounded by an autogenous matrix and is attached to a conditioned surface or more rarely just to each other.

Any natural or synthetic surface may be engaged. Translating this to the orthopaedic trauma setting, it refers to all tissues we handle, the surgical wound surface, and any foreign body, or fixation implant.^[18]^

The biofilm has a dynamic complex 3D structure, formed by the bacterial cells and their self-produced extracellular matrix (extracellular polymeric substance, EPS). The EPS represents >80% of the biofilm and consists of polysaccharides, glycolipids, cellular debris, enzymes, metal ions, and extracellular DNA.^[19]^ Macroscopically it has been described as looking like “slime” or “glycocalyx.” Mature biofilms are heterogeneous structures. Networks of ducts and channels have been described in vitro within the matrix, which allows the removal of waste products, the transport of oxygen, and other essential nutrients.^[20]^

The aggregated bacteria in a mature biofilm have passed into the sessile phenotypic state, are different from when they were free-floating, and had an active metabolism and fast replication (planktonic state). They are now in a stationary growth state, and much less metabolically active.

## 3 Biofilm epidemiology

The global impact of biofilm-related SSIs according to the National Institute of Health (NIH) and the Centres for Disease Control and Prevention (CDC) is huge. Biofilm SSI's account for up to 80% of the overall SSIs, and over 65% of the hospital-acquired infections (HAIs).^[21]^ Moreover, due to difficulties around their in-clinical diagnosis their prevalence and impact are considered to be under-reported.

It appears self-evident that these resilient infections will be associated with increased direct and indirect medical costs due to prolonged length of stay, need for sequential readmissions, surgical procedures, extended antibiotic therapies, and lengthy rehabilitation.^[21]^

Especially in orthopaedic surgery, where implanting devices into the human body is the norm, and since the propensity of biofilm formation on the surface of implants is so high, biofilm formation should always be suspected. Recent evidence has proven the presence of biofilms not only on fixation hardware, but also on grafts/soft tissue material used for cartilage, ligamentous, and tendon reconstructions.^[22,23]^ It has been reported that in general, implant-related SSIs are expected to increase significantly the next decades due to the increased demand for implant-related surgeries, the aging of the population and the higher prevalence of frailty among the operated patients, the obesity epidemic, and the raise of SSI risk factors.^[10,11,14]^

Both gram-positive and negative bacteria may create biofilms over the fixation implants, with most common bacteria including *Staphylococcus aureus*, *Staphylococcus epidermidis*, coagulase-negative *Staphylococcus*, *Streptococcus viridans*, *Enterococcus faecalis*, *Escherichia coli*, *Klebsiella pneumoniae*, *Propionibacterium*, *Peptostreptococci*, *Proteus mirabilis*, *Acinetobacter baumanii*, and *Pseudomonas aeruginosa*. There are certain fungal species (e.g., *Candida*) also that are most commonly associated with fungal or multispecies biofilms. Polymicrobial biofilms are observed in 10% to 30% in arthroplasty associated infections and even more commonly in fracture fixation SSIs.^[12,13,24]^

## 4 Pathogenesis

The creation and cycle of the effect of the biofilm can be described simplistically into the following 6 stages, which in reality represent a dynamic continuum.^[15,25]^

1. A conditioning film over the fixation implants is formed within seconds, consisting of blood products, platelets, sugars, albumin, fibrin, fibronectin/Laminin, and other extracellular matrix (ECM) proteins.
2. A mixture of free-floating/planktonic bacteria attaches to this conditioning film. In contrast to what was initially perceived, the implant's surface characteristics (as its roughness, hydrophobicity, and electrostatic charge) play a limited role. The bacterial attachment is to the conditioning film rather than the implant's surface per se. A family of bacterial adhesins (microbial surface components recognizing adhesive matrix molecules—MSCRAMMs) binds to the ECM proteins of the host. Each bacterial species expresses different numbers and types of adhesins.^[26]^
  The adhesion/attachment of the bacteria is initially reversible. As such, the microbes can still undergo random movements and can be still washed away and phagocytosed. Within minutes though, they become irreversibly attached due to the gradual secretion of more EPS.
3. After their irreversible attachment, the bacteria multiply and form microcolonies.
4. As the maturation of the biofilm progresses, more EPS is produced that reinforces the adherence of the biofilm.
5. Within the first hour from their irreversible attachment, bacterial homeostasis, and phenotype change. They express numerous new proteins, become less active, and fall in the sessile phenotype state.
6. Bacteria from the biofilm in a “quasi-sessile” state detach and reattach on other surfaces, a form of “metastatic seeding,” leading to infection chronicity, dissemination, and cross-contamination.^[25]^

In a similar but simpler scheme, the whole process of biofilm formation is described as in 3 phases: (a) aggregation = where mixed bacterial clusters form; (b) co-aggregation = when distinct bacterial clusters group together via interaction of specific receptors; and (c) co-adhesion = when co-aggregated bacteria adhere to the matrix and form the biofilm.^[27]^

The whole process of developing into a mature biofilm can be rapid and completed within 12 to 18 h.^[28,29]^

A number of authors have described biofilm in 2 layers (base and surface),^[30]^ containing bacteria with distinct roles, that is, “persisters” are the majority of slowly reproducing sessile bacteria; “wall formers” are responsible for maintenance of the physical barrier with the biofilm surface; and the “dispersers” are bacteria that are released to the environment.^[31,32]^ The dispersed bacteria transform into their planktonic state, developing appendices-like formations (fimbria, cilia, and flagella) that enhance their motility and give them a sense of “touch.”^[19,33]^

## 5 Biofilm functions

The biofilm acts as a physical protective barrier of the bacteria from the penetration of antibodies, bacteriophages, granulocytes and biocides, antiseptics, antibiotics, as well as environmental challenges such as dehydration, ultraviolet light, and low pH.^[34,35]^

It is generally accepted that the protective mechanisms that apply to the embedded bacteria are less relevant to genetic adaptations (i.e., mutations and acquired antibiotic resistance).^[36]^ They rather offer antibiotic tolerance, as a pure result of the phenotypic alterations, reduced metabolic activity,^[32]^ and a process called “drug-indifference.”^[37]^

Furthermore, the large numbers of sessile bacteria found within mature biofilms also contributes to the survivorship of some of them. These condensed colonies also facilitate horizontal genetic transfer (common in *Pseudomonas aeruginosa* biofilms) and the crossbreeding of resistance genes.^[38,39]^ Due to the same reason for hyperpopulation, rhamnolipids are produced by quorum sensing (QS, a form of intercellular signaling via autoinducer molecules), which offers anti-leukocyte defense.^[17]^ The hyperpopulation of embedded bacteria also creates conditions of starvation from nutrients. This leads to the expression of stress response genes (σ-factors) that protect the bacteria from antibiotics and host defenses. The lack of oxygen also leads some to undergo anaerobic metabolism, which contributes further to antibiotic tolerance.^[17,40]^

Following the autolysis of biofilm bacteria, the released eDNA (extracellular DNA) is concentrated within the EPS, which form chelating bonds with cations and can neutralize the activity of antibiotics such as tobramycin, as well as make the biofilm resistant to the effect of zinc, copper, and lead.^[30]^ Furthermore, interspecies interactions have been identified within some polybacterial biofilms, which offer enhanced biofilm protection to some antibiotics. This was reported for example when increased tolerance to vancomycin was observed, as the matrix of *Candida albicans* effectively shielded the coinciding *Staphylococcus epidermidis* within the same biofilm.^[41]^

A number of authors have defined these specific protective functions of the biofilm into the following 4 categories: (a) formation of a polymeric diffusion barrier to the antibiotics; (b) accumulation of antibiotic inhibitory molecules on the biofilm's outer surface; (c) expression of transport proteins or else called antibiotic efflux pumps that extrude the antibiotics; and (d) slow homeostasis that impairs the antibiotic uptake.^[42,43]^

The above mechanisms allow the biofilm bacterial population to survive the host defenses, as well as the standard antimicrobial treatment, which effectively addresses only the population of free-floating/planktonic bacteria. Characteristically it has been advocated that often the concentration of antibiotics needed for growth arrest of biofilm embedded bacteria is 1000 times higher.^[44]^ Moreover, the formation of the biofilms also contributes significantly to the chronicity and expansion of the SSIs, via their “metastatic seeding” to the surrounding area, but also via septic emboli to distant locations.^[25]^

## 6 Diagnosis

Unfortunately, the formation of biofilm does not only allow the embedded bacteria to evade host's defenses, or promotes antibiotic tolerance, but also makes the diagnosis of their presence exceedingly difficult.

The history of the suspected anatomical site can assist to identify risk factors or incidents during the postoperative period that may indicate the development of an SSI around the implanted fixation or graft material. Persistency despite pathogen-specific therapy, or the recurrence of an infection, especially if the isolated pathogens are the same, strongly indicates the presence of biofilm.^[17,44]^

The usefulness of biochemical inflammatory markers (such as the CRP, ESR, WBC) to the diagnosis of an SSI following orthopaedic trauma is limited. They mostly lack specificity and as they can be raised due to the inflammatory response to the initial trauma or the surgical hit, especially during the early postoperative period.^[45]^

The ultimate characterization of a Biofilm related infection (BRI), is the identification of bacterial communities enclosed in their matrix from samples taken from the surface of the implant. Clusters of such bacteria need to be evidenced with histological and microscopic findings (light or electron optic). The gold standard approach to diagnose any deep infection posttrauma is to take multiple tissue biopsies that need to be sent for culture and histopathological analysis.^[14,46]^ Similarly to diagnose a BRI, biopsies need to be taken from the implant's surface. Especially if the implant needs to be retained, or covers a large area, it is difficult to identify which areas are seeded. Acquiring representative samples of the bacteria is also challenging as the micro-organisms may lay deep within the matrix of the biofilm making their isolation difficult.

In vitro difficulties are mostly attributed to the low metabolic state of the embedded bacteria, which does not favor conventional culture-based methods. Thus, the results of the routine type of cultures are often false negatives (+30%), affected not only from the dormant state of the biofilm bacterial, but also compromised when local or systemic antibiotics were administered, as well as from the variable sampling technique at the time of biopsy.^[47]^ Novel culture strategies have been developed to address the accurate detection of BRIs overlying an implant. These obviously differ depending on the extraction or retainment of the implant per se.

When the implant is extracted, culture strategies mostly include the following:

- Vigorous multistage rinsing with phosphate-buffered saline (PBS) to remove host tissues debris, as well as planktonic or contaminating bacteria, and leave behind the mature biofilm with its embedded populations.
- The device is then swabbed and spread onto selective growth media or inoculated into liquid broth media. The challenge here is often choosing the areas of swabbing especially from a large implant.
- The agar encasement culturing method (AECM) can be a useful method to address this problem (for both aerobes and anaerobes). At the time of implant extraction, the device is encased in a thin film of melted agar. After the agar sets, bacterial colonies develop confirming the locations with biofilm. Digital photography is often used to determine the gradual colony outgrowth and map the different areas of the device. A similar technique is the full immersion into melted agar growth media, which after the incubation period, declares the areas of biofilm as areas of colony outgrowth. It is mostly used in small-sized extracted devices.^[48]^
- The need for the surface recording of the suspected areas can be also addressed with the use of fluorescent probes that stain rRNA of living bacteria or other proteins (fluorescent in situ hybridization, FISH). These probes can target specific nucleic acid sequences of specific bacteria or also target components of the EPS. After staining, fluorescent microscopy or confocal laser scanning microscopy (CLSM) allows the identification of embedded specific bacterial species. However, this technique cannot exclude the escape of other species from detection that do not react to the specific probes, nor can fully differentiate host matrix proteins from those of the EPS.
- A number of clinicians are using blood culture bottles to inoculate and culture periprosthetic tissue samples. This has been proven to have diagnostic accuracy similar to cultures of sonication fluid.^[49]^
- The sonication represents basically the use of ultrasound low-frequency waves to an implant immersed in a fluid (sonication bath). The extracted implant is first rinsed with sterile PBS to remove debris, then is placed in a sterile container filled with saline, so that the sample is ready on arrival to the laboratory. The sonication lasts usually 5 min. The ultrasound waves cause disruption of intercellular links, as well as deagglomeration of the adhesins that eventually dismantle the matrix and release the sessile bacteria.^[47]^ The dislodgement of the bacteria from titanium or stainless steel surfaces has been proven to be much higher with sonication than via scraping with a scalpel, rinsing, tissue grinding, etc. Further disruption of the biofilm matter is reported when a vortexing step is added after the extraction of the sonication fluid and prior to its culture. Direct cultures of the fluid, on selective growth media and lengthy molecular and microscopic analysis of them, follow.

A significant increase of positive cases has been reported with sonication to >30% when compared to tissue cultures. Furthermore, the speed of getting culture-positive results is quite higher (63% of positive results are given within 48 h).^[50]^ The sensitivity of culturing the sonicate fluid has been reported to reach over 85% versus 65% of the tissue cultures, and even 100% when the sonication fluid was inoculated in blood culture bottles and cultured for 14 days.^[49,50,51]^

- In the absence of sonication, the use of chemical agents to dismantle the biofilm (as dithiothreitol, DTT), or mechanical grinding of the extracted samples into containers with 1 mm beads, has been advocated. Such measures have been shown to improve the accuracy in the presence of BRIs.^[52,53]^
- Nonculture techniques for detecting the bacteria have also been utilized. The sensitivity of histopathology for bone infection remains low (>50% on some occasions).^[54]^ Scanning electron microscopy (SEM) can identify with its high resolution the biofilm matter and even screen the surface characteristics of single bacterial cells. However, it is often not practical due to the size of the samples to scan all surfaces, while dehydration through processing can alter the features and hinder the diagnosis.
- Polymerase chain reaction (PCR) and DNA sequencing are also reliable methods used as adjuncts or when the sample is small (i.e., tissue samples from the vicinity of non-extracted implants, or samples with small numbers of bacteria). The sampled bacterial DNA or RNA is probed with primers (small nucleic acid sequences that target specific regions of the genetic code) and then undergoes amplification that allows its detection. Problems may arise when antibiotic treatment has preceded (false negatives) or when contamination of the sample at acquisition or processing occurs (false positives). The strengths of the method are that it can detect even small amounts of bacterial genetic material, whereas using reverse transcriptase to amplify mRNA, the inaccuracies from detecting material of dead bacteria rather than active can be tackled.
- Biomarkers have also been explored in an attempt to augment the accuracy of diagnosis of SSIs with orthopaedic implants. The detection of alpha-defensin (an innate antimicrobial peptide) has recently evolved to a point-of-care test,^[55]^ from the originally described more complex enzyme-linked immunosorbent assay (ELISA).^[56,57]^ A randomized trial (ClinicalTrials.gov Identifier: NCT02868736) is at its recruitment phase, investigating the effectiveness of either of these 2 methods of detecting this potentially useful biomarker (ELISA vs lateral flow test).

All these diagnostic techniques are problematic and less useful when the implant is retained, which is a common strategy with fracture fixation related SSIs. When the implant is not extracted, the laboratory diagnosis utilizes advanced culture and nonculture techniques described above, but the confirmation of a BRI becomes certainly more difficult. Prolonged cultures for even more than 14 days, employment of PCR, and modern biomarkers are often employed.^[14,58]^

## 7 Treatment

In orthopaedic elective surgery, the time interval between the index surgery and the onset of clinical symptoms, their severity, and the state of the host, are usually enough to guide clinicians to an estimation of the route of infection and to plan their management strategy.

From an orthopaedic trauma surgeon's perspective, a number of additional factors are important, starting with the first suspicion of a potential BRI. Signs of a stable bone-fixation construct, in an overall well-reduced fracture, absence of radiological signs of loosening of the hardware, should all be ascertained before a decision is made. The obvious dilemma in most cases, is between implant retention and suppression of the infection until fracture union with almost the certainty of developing biofilm and chronic osteomyelitis, increasing, therefore, the difficulty of achieving final eradication of the infection, vs. the early removal of all implants, attempting to eradicate the infection first and proceeding to secondary reconstruction in an aseptic environment (a more common practice in elective orthopaedics).^[14,46]^

In the presence of a healed fracture, or of an unstable osteosynthesis, of malreduction, or when the host's response to an initial retention/suppression strategy fails, the decision is relatively easy. Usually, in a multistage protocol, the following steps occur:

1. thorough debridement with removal of all foreign material.
2. exchange to an external bridging fixation.
3. plus/minus application of a local spacer loaded with antibiotics.
4. plus/minus soft tissue reconstruction in case of large defects.
5. laboratory testing of multiple samples (often including the extracted implants); adequate pathogen specific antibiotic therapy.
6. followed by definitive reconstruction of the affected area.

This is the main domain of advanced limb reconstruction techniques including the induced membrane technique, circular frame fixators, and distraction histogenesis.^[59,60,61]^

In either of the 2 strategies (debridement/retention/suppression vs. debridement/removal/eradication), the presence of biofilm creates additional difficulties. It is well established that the longer period of time mature biofilm is present in an area, the higher the risk for the BRI to become recalcitrant, leading to failures and recurrence of the infection.^[38]^

If implant retention is chosen, the assumption that biofilm will be created if not already present should be made. The shortest possible time period between the initial implant insertion, the manifestation of SSI signs, and the initial local debridement, is necessary. If the diagnosis is at the early stages post initial fixation, then initial debridement to minimize the bioburden is adequate. If the technique of tissue biopsy is meticulous and prompt, then antibiotic pathogen-specific treatment can start early and the chances of successful suppression of the BRI are higher. At the same time, it is understood that in the retention cases, visualizing and approaching in situ all surfaces where the biofilm may be located is impossible. Some residual biofilms will be left behind. The host defenses will not be able to invade, whilst standard antibiotics, even if specific to the particular pathogens, should be expected to be less effective to the biofilm colonies.^[12,58]^ This should be clearly described to the patients, and prepare them for a potential failure of the retention/suppression strategy.

There are further measures that can be undertaken to optimize the chances of successful suppression until union with retention, which include the utilization of local antibiotics, or compound schemes that have activity against biofilm bacteria (i.e., Rifampicin, Colistin, Daptomycin, Linezolid, Ceftaroline).^[62,63]^ Rifampicin is an antibiotic that penetrates the biofilm and is a cornerstone drug for staphylococcal/streptococcal BRIs, never thought of as a monotherapy (bacteria develop very rapidly resistance to it).^[64,65]^ Colistin is an antimicrobial peptide that has demonstrated its effect in longstanding infections of multiresistant gram-negative bacteria, and has antibiofilm action when combined with Fosfomycin.^[66]^ The duration of antibiotic suppression may vary depending on the specific species sensitivities, the host response and progress of inflammatory markers, as well as the progress of fracture union, which eventually may allow removal of all implants and the attempt of eradication. The usual period of continuous suppression is within 6 to 12 weeks with close biochemical and radiological monitoring.^[14,46,58]^ In fungal BRIs the use of amphotericin and echinocandins were shown to be better than the azoles in the presence of biofilm. In general fungi biofilms are considered very difficult to eradicate and removal of all implants is the recommended strategy.^[67,68]^

At a more experimental level, additional strategies have been tested, aiming to destroy biofilm in situ, that is, use of citric acid as a cytotoxic agent, bioactive enzymes (as dispersin B), electrical stimulation, pulsed electromagnetic fields, and treatment over the retained implant with promising results.^[69,70]^ Bacteriophages (viruses targeting the bacteria) have failed to show their effectiveness in vitro mostly due to mutations and adaptation of their targets.^[71]^

The importance of thorough debridement in all SSIs, especially in more chronic infections is paramount. Mechanical debridement of the wound, of the bone and implant surfaces (if a retention strategy is chosen), excision of necrotic tissue and avascular fragments, but also of the “slimy material” underlying a plate or surrounding a nail, is essential. When the eradication of the infection is the aim (last stage of retention strategy after fracture union or at all stages of removal of implant strategy) all foreign material is removed, the canal is over-reamed, the screw holes/exfx pin sites are over drilled and all debris is thoroughly washed out. The role of special devices as the Reamer Irrigator Aspirator (RIA) can offer additional advantages for intramedullary areas.^[72,73]^

Insertion of local antimicrobials and antibiotics after the debridement, has also been shown to be effective. It allows the delivery of very high doses of these agents to the infected area, at levels that they can affect the sessile bacteria biofilm populations. Minimal systemic toxic effects are recorded, which would be apparent if these doses would be administered via other routes.^[74]^ Several studies have documented that to inhibit or kill biofilm embedded bacteria, the minimal inhibitory concentration 90 (MIC90) has to be 100 to 1000 times higher than the effective MIC90 threshold for free-floating bacteria.^[44]^ Local agents should be considered both when all implants are removed and bony defects are created, but also when a retention strategy is chosen. Managing the dead space with the insertion of antibiotic-loaded spacers (non-absorbable PMMA based or absorbable calcium sulfate/phosphate substitutes), as well as delivering high doses of these agents (most commonly aminoglycosides, vancomycin, cefuroxime, antifungals) have been widely adopted.^[14,58,74]^

The effective management of these apparently complex clinical scenarios can only be achieved with the prompt engagement of appropriate multidisciplinary teams. From the early phase of initial presentation and diagnostics, to the crucial phase of choosing the appropriate strategy between retention/removal of the implant, till the completion of the postoperative follow up, the input of orthopaedic surgeon, plastic surgeon, microbiologist, and radiologist is very important. The surgical strategy should be on parallel to specialist efforts of optimizing the general state of the host, adequate nutrition, control of diabetes, cessation of smoking, adequate soft tissue coverage, and optimization of peripheral blood supply.^[14,17]^

## 8 Prevention

Benjamin Franklin in his short essay on how to prevent domestic fires, expressed one of the most standard concepts in medicine: “an ounce of prevention is worth a pound of cure.” A biofilm-related infection especially in orthopaedic trauma is one of the most challenging complications to diagnose and manage. For this reason, significant effort has been put recently into identifying prevention measures to its formation.

General measures of preventing SSIs (implant sterilization, clean air systems, and reduced traffic in the operating theatres, skin antisepsis, perioperative use of antimicrobial prophylaxis, careful draping, meticulous surgical technique, maintaining intraoperative normothermia, and good oxygenation) are of paramount importance and should be observed in all orthopaedic trauma procedures.^[75,76]^

Vaccination strategies aimed at the *Staphylococcus aureus* species, one of the most common pathogens associated with SSIs and BRIs, have been developing for more than 50 years now, unfortunately without any significant clinical impact as yet. The results of large-scale trials (like the STRIVE - ClinicalTrials.gov: NCT02388165 on a 4-antigen vaccine has been completed earlier this year) may boost their use.

The prevention of bacterial colonization and biofilm formation in particular on the implanted fixation devices has attracted additional interest over the past 2 decades. Variation of the implant characteristics by using different alloys (titanium, stainless steel), modifying surface characteristics (roughness, porosity, physico-chemical composition, polarization), as well as surface coatings have all been tested.^[77]^

Different alloys have shown a different affinity to bacteria mostly due to some of their components and their surface roughness.^[17]^ Vanadium-free titanium alloys are suggested as the least attractive for bacteria, however, a number of clinical studies have failed to demonstrate this as an independent risk or protective factor in orthopaedic trauma SSI.^[78,79]^

Treatment technologies of the outer surface of the implants as thermal cycling, modification of the crystalline structure of titanium oxide layers or the ultraviolet (UV) irradiation on titanium dioxide-coated implants, or the creation of an hydrophobic environment have been shown to inhibit bacterial adhesion.^[80,81]^ Passive surface finishing/modification (PSM) allows modifications of the surface chemistry and structure. Especially for non-ingrowth implants (the vast majority of what is used in orthopaedic trauma but not in elective orthopaedics) these methods can be cost-effective and relevantly simple in reducing the ratio of BRIs. As proven, however, the bacterial adhesion is happening to the conditioning film, rather than to the implant surface per se, which significantly decreases the effect of this or similar passive surface strategies on actual protection.^[25,29]^

Coating technologies of fracture fixation implants have been attempted with numerous agents. The use of polymers, hydrogels or amphiphilic compounds (i.e., polymethacrylic acid, polyethylene oxide, polyethylene glycol, cross-liked albumin, biosurfactants, etc), to organic (peptides, cytokines, chitosan, antiseptics) and inorganic molecules (iodine, selenium, carbon-based nanotubes), metal ions (silver, zinc, copper), and of course antibiotics has been proven challenging. Combinations in multiple layers of different agents (smart coatings) have also been attempted aiming to control time period of their effect as well as combine different mechanisms of action.^[82]^ Since most commonly the colonization and biofilm formation is rapid, the antimicrobial effect should be exerted immediately at implantation and at least endure for the first few days.^[80]^ Coatings can be divided into degradable, nondegradable, drug-eluting, and contact killing. Most are incorporated into the implants at the phase of production, using complex new technologies. The option of custom-made coatings, which can be loaded to the implant intraoperatively has been also explored.^[83]^ Biocompatible hydrogels able to deliver high concentrations of different antibacterials (aminoglycosides, fluoroquinolones, glycopeptides) exerting their effect for 72 h, have been released in Europe.^[84]^ The main advantage of the latter, is their increased applicability to different anatomical sites and to already existing fixation devices.

These coatings, in addition to having clear antibacterial and antibiofilm properties, should also demonstrate a safe profile to the patient, allow fracture healing processes, be easy to implement, and have an affordable cost. The regulatory approval of these pharmacologically activated fixation devices is a challenge on its own, as the implant becomes similar to a drug agent with difficult to determine long-term effects.

## 9 Conclusion

Biofilm-related infections will increase in the near future, together with the challenges of our surgical specialty, which relies largely on the implantation of fixation implants, joint arthroplasties, foreign materials, and grafts. This group of surgical site infections is the most difficult to diagnose, to suppress, to eradicate, and in general to manage. Multispecialty teams involved in all stages of care are an effective way to improve results, save resources, and time for the sake of our patients and the health system. Significant steps have occurred recently to the prevention and development of clever tools that we can employ in this everlasting fight with bacteria.
